# Supplementary material for: Comparing Viral Vectors and Fate Mapping Approaches for Astrocyte-to-Neuron Reprogramming in the Injured Mouse Cerebral Cortex
Source: Cells. 2024 Aug 23;13(17):1408. doi: 10.3390/cells13171408 (PMC11394536; doi:10.3390/cells13171408)
Supplement: Supplementary file 1 [file cells-13-01408-s001.zip › cells-3161358-supplementary.pdf]

Supplementary Figure S1

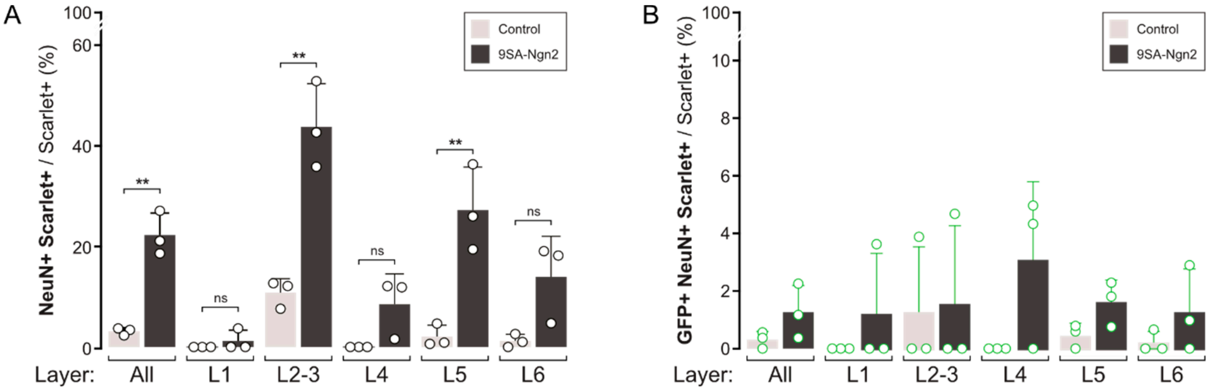

Figure Supplementary S1 – Distribution of the endogenous neurons labelled by AAVs across the cortical layers

A Layer distribution of the NeuN+ mScarlet+ cells observed after the injection of Control (light grey) or the 9SA-Ngn2 construct (dark grey) in GFAP::Cre/GFPrep mice. **B** Distribution across the cortical layers of the few GFP+NeuN+mScarlet+ cells. No statistical difference could be observed between Control (light grey) and mice injected with the 9SA-Ngn2 construct (dark grey). Plotted data represent the mean±SD of three biological and technical replicates. Statistical differences between the two conditions have been calculated by unpaired t-test. **: p-value < 0.01; no label or n.s.: no statistically-relevant difference.

Supplementary Figure S2

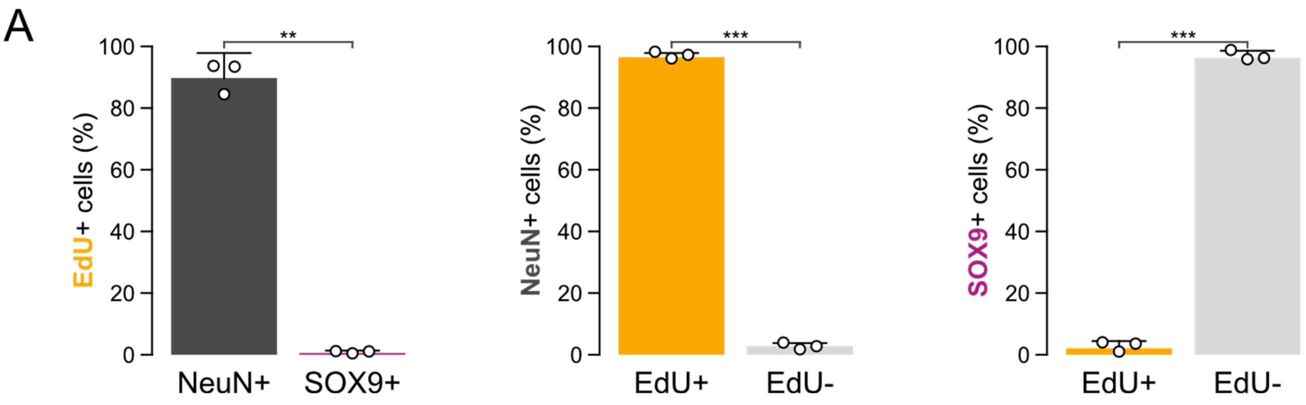

Figure Supplementary S2 - FLEX-AAVs injection in Cre-negative mice

A Quantification of the labelling specificity (left) and sensitivity (middle and right) of endogenous neurons birthdating with EdU. Specifically, the graph on the left reports the fraction of EdU+ cells

that express the neuronal marker NeuN+ or the astrocytic marker Sox9. The plots in the middle and right respectively report the fraction of all NeuN+ and Sox9+ cells that are labelled by EdU. Every dot represents the figures obtained from one section of one animal. Statistics have been calculated by paired t-test. **: p-value < 0.01; ***: p-value < 0.001;

Supplementary Figure S3

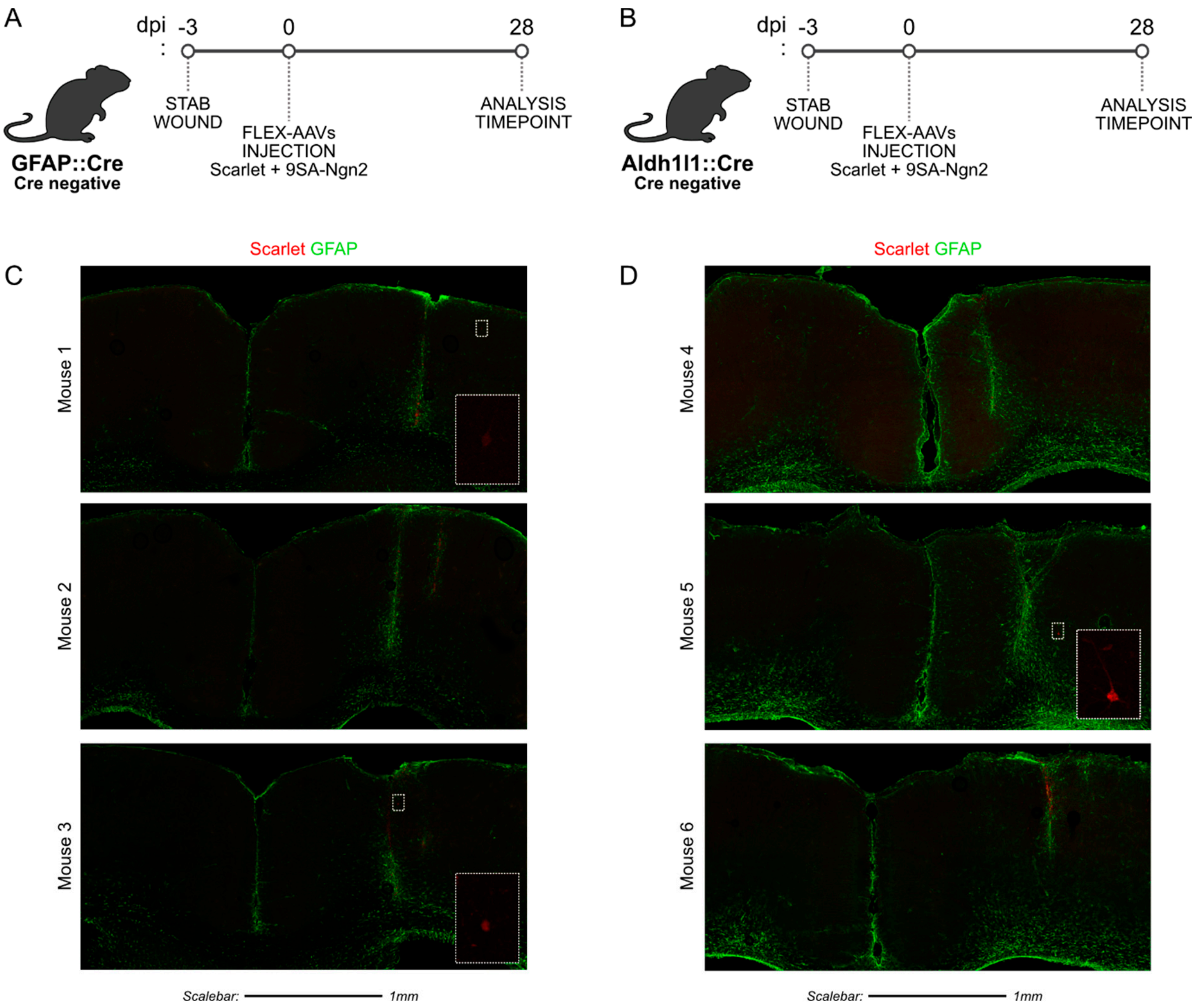

Figure Supplementary S3 - FLEX-AAVs injection in Cre-negative mice

A Cre-negative mice from two different astrocyte-Cre transgenic litter received a cortical stabwound and intracerebral injection of reprogramming FLEX-AAVs (mScarlet + 9SA-Ngn2) three days later. Cre-independent expression has been investigated by immunohistochemistry 28 days after the injection (28dpi). **B-C** Confocal pictures of a 20 µm stack of the SW-injured cortex of Cre-negative GFAP::Cre (B) and Aldh1l1::Cre (C) mice 28 days after the injection of FLEX-AAVs. Reactive

astrocytes around the SW express Gfap and rare cells express mScarlet in a Cre-independent manner. Scalebar 1mm

Supplementary Figure S4

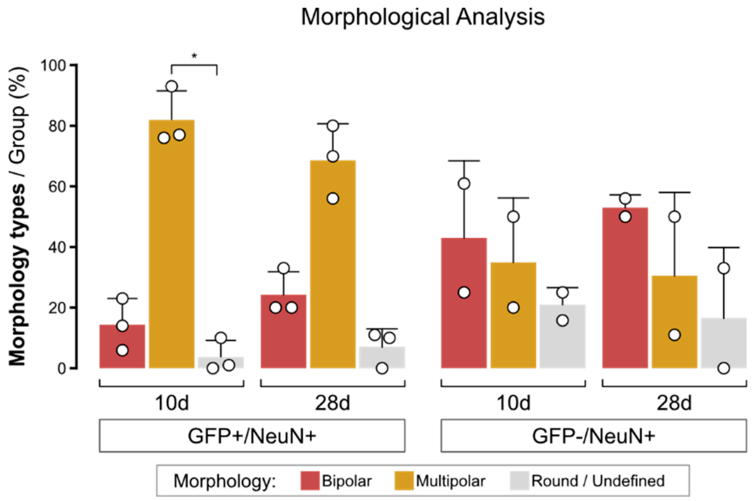

Figure Supplementary S4 – Morphological types of cells observed after Mo-MLVs injection

Cells infected by Mo-MLVs (all Scarlet+) have been classified according to the expression of NeuN and GFP, and their morphology. Cells extending one long process or with few long processes mostly directed towards opposite side of the soma have been classified as "Bipolar", cells having equally long processes directed in all directions have been classified as "Multipolar", while cells without clear processes have been classified as "Round/Undefined". Plotted data represent the mean±SD of two or three biological replicates: each white dot represents one animal for which data from 1 to 3 sections have been averaged, according to the presence of cells. For example, no GFP-NeuN+ cells could be observed in any of the three sections (technical replicates) in one of the three animals sacrificed at 10 dpi and a different one at 28 dpi (biological replicate). These two mice were excluded from the plot. Statistical differences between the two conditions have been calculated by Kustal-Wallis test. *: p-value < 0.1; no label: no statistically-relevant difference.
